# Medical School Students’ Preferences for and Perceptions of Teacher Written Corrective Feedback on English as a Second Language Academic Writing: An Intrinsic Case Study

**DOI:** 10.3390/bs13010013

**Published:** 2022-12-23

**Authors:** Barry Lee Reynolds, Xiaofang Zhang

**Affiliations:** 1 Faculty of Education, University of Macau, E33, Av. da Universidade, Taipa, Macau SAR, China; 2 Centre for Cognitive and Brain Sciences, University of Macau, Taipa, Macau SAR, China

**Keywords:** writing feedback, teacher feedback, direct feedback, indirect feedback, grammar feedback, content feedback

## Abstract

This intrinsic case study investigated English as a foreign language (EFL) medical students’ preferences for and perceptions of teacher written corrective feedback (WCF) on their academic writing. Chinese-speaking second-year first-semester undergraduate medicine majors (*n* = 71) enrolled in an academic EFL “reading to write” course at a university in northern Taiwan were recruited as participants. Qualitative content analysis, as well as some descriptive statistics, was used to investigate data gathered from participants’ responses to an open- and closed-ended questionnaire. The questionnaire enquired about their preferences for and perceptions of teacher WCF relating to writing structure, writing content, and writing mechanics. Qualitative content analysis of two in-depth semi-structured interviews with the English teacher uncovered why the participants preferred certain WCF types and perceived them as helpful. Questionnaire data revealed that students showed a preference for WCF relating to writing structure over content and mechanics, and direct feedback over indirect feedback for both writing content and structure. Compared to writing structure and writing content, the examples given by students of the most (*n* = 25) and least helpful (*n* = 14) feedback were predominantly related to writing mechanics. The interview transcript data underscored the influencing factors of EFL medical students’ preferences and the perceived benefits and challenges related to feedback. These findings suggest that writing teachers should consider the specialized preferences of particular learner groups (e.g., EFL medical school students) prior to administering feedback.

## 1. Introduction

Written corrective feedback (WCF) is an important facet of foreign language pedagogy and has been extensively investigated in the field of EFL writing [1,2,3]. WCF, a response to linguistic errors in students’ writing, is frequently provided in pedagogical settings by educators, peers, or automated computer systems; it is sometimes provided by native speakers and foreign language learners in everyday contexts [3].

Since Truscott [4] initiated a debate on WCF’s effectiveness, there has been growing evidence that WCF can assist writing accuracy improvement, both immediately and over time [5,6]. However, such improvement seems to be related to linguistic factors, e.g., feedback and error types, and also to learners’ perceptions of and preferences for WCF types, especially some specific affective factors such as positive emotions [2,6]. Learners’ affective engagement with WCF can affect how they perceive and react to it [7]. Previous studies have identified different WCF types [1,8,9]. Specifically, indirect WCF appears to be the most frequently used type in real classroom settings, but is less preferred by students with lower language proficiency [1,9,10]. Instead, students preferred to receive direct WCF from their teachers [11,12]. Students with either high or low language proficiency preferred WCF on their grammatical rather than lexical or mechanical errors [13,14]. In summary, learners can benefit from WCF if teachers’ teaching practices match their preferences [15]; such preferences are often related to WCF types and learners’ language proficiency level. Therefore, exploring learners’ preference for WCF type may facilitate a better understanding of the role of WCF in EFL teaching and learning.

In addition to its general EFL relevance, WCF has long been an important topic for medical educators [16,17]. However, few studies have focused on teacher WCF on EFL medical students’ English writing. Similar to EFL students, EFL medical students’ perceptions and preferences represent key variables influencing the role of WCF; any disconnection between students’ understanding and teachers’ expectations can impair learning effectiveness [1,10]. The current literature, particularly relating to WCF, is limited to certain writing genres (e.g., descriptive, narrative, and journal writing) [18]. Therefore, WCF in different instructional contexts needs to be further explored. Furthermore, while researchers have examined students’ and teachers’ reasons for their WCF preferences and discovered important discrepancies—in terms of both how and why WCF should be provided [1]—the way in which teachers’ and learners’ beliefs about WCF converge and diverge needs a more systematic examination [1]. This intrinsic case study considered how individual factors interacted with elements in the instructional context as well as the broader social context in which feedback was provided; this perspective seems to be missing in previous studies. Therefore, this intrinsic case study will deepen understanding of how and why EFL learners respond to WCF in the Taiwanese context. We selected the Taiwan EFL medical school learning context because this unique population of students are of particular interest to our research aims, namely, attempting to understand a unique situation in the Taiwanese EFL learning context. As described above, we are interested in investigating EFL medical students’ preferences for and perceptions of teacher WCF on their academic writing. Although attention to writing is increasing in the Taiwanese EFL context, the unique writing needs of EFL medical students may not be receiving adequate attention. We investigated this specific group as they face limited writing instruction and their specific future writing needs were not necessarily being met.

Addressing these gaps, this study investigated EFL medical students’ perceptions of and preferences for teacher WCF on their English academic writing, based on an intrinsic case study. As an intrinsic case study, the focus is on the case itself rather than an overarching theory—the case presents a unique situation in the Taiwanese EFL learning context [12]. We have a genuine interest in this case, which helped us address the following research questions:

RQ1: What types of WCF do EFL medical students prefer to receive?

RQ2: What types of WCF do EFL medical students perceive as helpful?

RQ3: Why do EFL medical students prefer certain types of WCF?

RQ4: Why do EFL medical students perceive certain types of WCF as helpful and others as unhelpful?

## 2. Literature Review

### 2.1. WCF

WCF is highly complex, as reflected in controversies surrounding such issues as “whether to correct, what to correct, how to correct, and when to correct” [8], p. 26. As a very researchable field with both theoretical and pedagogical significance, WCF studies have increased dramatically over the last three decades [7,19]. Theoretically, cognitive interactionist theory [20] claims that corrective feedback facilitates language learning by assisting learners’ establishment of “target-like form-meaning mappings” [7], p. 336, when communicating with other people. Additionally, skill learning theory [21] affirms that corrective feedback can help learners consolidate their declarative knowledge of the foreign language. Pedagogically, researchers enquire whether WCF helps second language (L2) learners improve their writing effectiveness [22]; they have also focused on improving teaching pedagogy [7].

WCF can be divided into three major types: direct, indirect, and metalinguistic [8]. Direct WCF, the most explicit type, refers to overt correction of errors and provision of correct forms [23]. Indirect WCF, the least explicit, identifies errors without supplying correct forms [23]. Although metalinguistic WCF shares some features with indirect WCF, they differ because the former explains the nature of the errors to encourage learners’ self-correction, while the latter merely identifies errors [7]. Specifically, metalinguistic WCF can either provide an error code to indicate specific errors or a brief metalinguistic explanation [24]. Previous literature has shown an increasing interest in investigating whether more explicit WCF types facilitate language learners’ L2 development (e.g., [24,25,26]). In this study, our investigation of EFL medical students’ preferences for and perceptions of direct and indirect WCF on their academic writing aimed to further explore the complexity of WCF and contribute to the growing literature in this area.

### 2.2. EFL Students’ Preferences for and Perceptions of WCF

WCF contributes to both learning and teaching; its effectiveness is related to learners’ preferences for and perceptions of WCF types relating to a variety of language errors. Thus, these preferences and perceptions have been investigated [2,8]. Previous studies have shown that learners have a strong demand for WCF and that mismatched learner–teacher perceptions of WCF may obstruct learners’ effective usage of WCF [27]. However, few studies have explored learners’ preferences for WCF types (e.g., [1,15,28,29]). Specifically, in comparison to overt correction of lexical errors, learners preferred WCF based on error codes [29]. Rummel and Bitchener [15] compared the effectiveness of direct and indirect WCF on grammatical accuracy and found that most learners preferred to receive indirect WCF. Amrhein and Nassaji [1] investigated the preferences of ESL students and teachers in terms of WCF for writing accuracy. Although similar opinions were shared by both ESL students and teachers, the researchers found that ESL students preferred teachers to provide WCF on all errors, but teachers preferred to offer selective WCF [1]. As in previous WCF studies, this intrinsic case study focuses on students’ preferences for and perceptions of WCF; however, unlike previous studies, we investigated the specific context of EFL medical students. The findings have the potential to inform writing teachers in Taiwan about EFL medical students’ feedback preferences, and enrich the WCF literature with findings about a specific writing genre.

## 3. Methodology

### 3.1. The Case

#### 3.1.1. Participants

L1-Chinese-speaking second-year first-semester undergraduate medicine majors (*n* = 71) who enrolled in an academic “reading to write” course (taught by one of the authors) at a university in northern Taiwan were recruited. We followed the TESOL International Association’s research guidelines https://www.tesol.org (accessed on 8 September 2020). The research purpose was clearly explained to all participants; they were also informed about privacy, i.e., that their anonymity would be protected throughout the research process. Additionally, researchers explained that participation was voluntary and that participants could withdraw at any time without penalty. Participants were aware that the research results would be used in academic publications and were told to contact the researchers if they had further questions. Before participation, a signed informed consent form was obtained from each participant confirming their understanding of these issues.

The teacher possessed the terminal PhD research degree in education and a practitioner-oriented master’s degree in teaching English to speakers of other languages. At the time of data collection, the teacher had accumulated 13 years of experience in teaching academic English reading and writing courses. However, only 2 out of the 13 years were in a context where the students were medicine majors. The “reading to write” course considered in this study was the second time the teacher had taught this course to medicine majors.

#### 3.1.2. The Course

Participants were given two hours of instruction by the same teacher once a week for 18 weeks, and wrote four essays (between 800 and 1200 words) on topics related to global health. Regarding lesson design, academic reading and writing skills were usually taught in a lecture format during the first hour of class and investigative group work was conducted during the second hour, in which students practiced the skills they learned in the first hour. The teacher provided WCF on the students’ writing. The course aimed to improve students’ understanding of written English and receptive and productive academic vocabulary knowledge and inculcate academic reading and writing skills, thereby preparing students for satisfactory performance, at the very least, in English-taught academic courses and cultivating a love of reading and writing. The students practiced specific strategies for academic reading and writing. 

The reading skills were imparted to the students using a textbook during intensive reading lessons. The teacher would name the skill being practiced, then use the textbook activities and articles to provide examples that would be completed together as a class and displayed on presentation slides. Thereafter, the teacher would ask the students to put these skills to practice when reading academic journal articles. The teacher would ask the students to use highlighting and notetaking in the margins to show proof of the implementation of the reading skills. While some of the reading was done in class, the remainder was finished out-of-class. As the teacher had assigned these articles to the students for reading, the teacher could easily check whether the students were able to adequately apply the skills for reading academic journal articles. The reading exercises were necessary for the subsequent completion of the writing assignments. 

For teaching writing skills, the teacher scaffolded the students through the completion of incremental writing activities from the textbook. For example, the textbook provided guidance on how to construct main idea sentences and support these sentences with details. The textbook also provided activities on how to structure an argument and use academic vocabulary to express one’s views. The teacher guided the learners to complete these activities individually and displayed them on presentation slides. Thereafter, the learners were asked to get into groups for scaffolded discussions on the topic of the writing prompts. The teacher would move the discussion along, asking students to engage in answering specific questions that aimed to activate their background knowledge and generate opinions on the topics. During these discussions, the teacher would interact with the different groups to confirm and, if necessary, clarify their understanding of the writing prompt. The students were also expected to search academic databases linked from the university library to obtain journal articles relevant to the topics of the writing prompts. These articles would be used to support the claims the students made in their academic writing. In other words, the structure, idea generation, and research stages of writing were completed in groups during class, while the writing and editing were completed outside the class. 

The readings were accompanied by associated reading, writing, and vocabulary-building activities. The students experienced similar challenges in academic reading and writing assignments as they would encounter in English-taught academic courses. The reading materials were both textbook-based and academic journal articles related to global health. It was expected that by the end of the course, the students would be able to achieve the following:

- Activate background knowledge relevant to the content of academic texts;
- Preview the content and organization of texts before starting in-depth reading;
- Read actively by asking themselves questions and monitoring comprehension as they read;
- Focus on understanding the main ideas in paragraphs;
- Read for details to establish connections among them and the main ideas of texts;
- Practice new reading, writing, and vocabulary-building skills and strategies;
- Learn academic vocabulary, especially those from the academic word list (AWL);
- Understand the meaning of unknown words encountered in texts by using their surrounding context;
- Focus on the meaning of key words in texts by connecting their meaning to familiar words and phrases;
- Recognize different word forms to quickly and efficiently increase receptive vocabulary knowledge;
- Paraphrase texts;
- Engage in thoughtful, reflective, and independent thinking to make sense of texts;
- Write main idea sentences and detail sentences to support those main ideas;
- Undertake some research on global health topics;
- Develop a deeper understanding of global health topics and become adept at using global health vocabulary to write about these topics;
- Critically discuss global health topics;
- Write academic essays on global health topics;
- Engage in dialogue journal writing with the teacher to increase writing fluency;
- Recognize the cohesion of vocabulary, structural features, and organization of global health research articles.

#### 3.1.3. The Context

In recent years, Taiwanese universities have adopted a policy requiring students to pass the General English Proficiency Test (GEPT) or an equivalent international examination to meet one of the graduation requirements [30]. GEPT is designed and administered by Taiwan’s Language Training and Testing Center (LTTC); it aims to provide an effective measurement of competence for English learners (including both students and the general public) at five levels (Superior, Advanced, High-Intermediate, Intermediate, and Elementary). An increasing number of universities now require their students to demonstrate their English proficiency by passing either the Intermediate or High-Intermediate level, suggesting that the GEPT has positively affected English learning in Taiwanese higher education. All five levels assess the four language skills of listening, reading, writing, and speaking; tests contain two sections—the first measures receptive English knowledge (listening and reading), while the second tests productive English knowledge (writing and speaking). As the test levels increase, writing is given increasing weight; thus, writing is often highly valued by teachers and students [31]. Nevertheless, “English writing instruction in Taiwan has not matured and there is much room for improvement” [32], p. 12. Additionally, whereas most EFL students only focus on passing the GEPT test and have limited needs for English writing, EFL medical students have different learning goals and learning environments; they need to learn about other types of writing, especially academic writing, to further their medical careers. Nonetheless, the teaching of writing is minimized in Taiwan’s medical EFL university language learning contexts. Therefore, this learning context is characterized by an imbalance between teaching policy and actual teaching practice. Against this background, the current study’s findings can make significant contributions, as they provide suggestions for improving EFL medical students’ English writing through WCF and related pedagogical implications.

### 3.2. Research Instruments

The research instruments (the questionnaire and interview protocol) were developed based on the typology of WCF [33]. Thereafter, an L2 writing researcher with extensive experience in the teaching of L2 writing was consulted for a quality check of the two instruments. 

#### 3.2.1. Open- and Closed-Ended Questionnaire

An open- and closed-ended questionnaire (Appendix A) was constructed to collect information on learners’ background characteristics (age, gender, English proficiency level, and learning experience) and preferences for and perceptions of teacher WCF on writing structure (i.e., topic sentences, supporting sentences, closing sentences, transition, spelling, capitalization, punctuation, and formatting), writing content (i.e., organization of ideas, concepts, tone, vocabulary use, and grammar), and writing mechanics (i.e., spelling, punctuation, and formatting). As part of this, participants were asked to provide examples of the most and least helpful kinds of feedback received. However, while there were 71 participants, not all provided answers to all of the questions.

#### 3.2.2. In-Depth Semi-Structured Interviews

Two follow-up in-depth semi-structured interviews (Appendix B) were conducted with the EFL teacher (one of the authors) to investigate why EFL medical students preferred and perceived certain types of WCF as helpful. Both interviews were conducted in English (the EFL teacher’s native language). The aim of the first interview was for the EFL teacher to elaborate on and interpret some of the students’ responses to the open- and closed-ended questionnaire. The second interview was conducted after data analysis of the questionnaire and first interview, to gain an overview of how and why EFL medical students respond to WCF in the specific Taiwanese context.

### 3.3. Data Analysis

We employed a mixture of top-down coding, which applies preconceived codes based on the research focus and research questions, and bottom-up coding, which extracts codes from the data [34]. RQ1 and RQ2 were mainly addressed through the questionnaire data, which were analyzed using focused coding. This involved identifying and re-examining specific codes before developing different categories. This enabled us to report some descriptive statistical results by summing responses to questions 1, 2, 3, and 4 (Appendix A). RQ3 and RQ4 were mainly addressed through semi-structured interview data. The researchers first transcribed the interviews verbatim into NVivo 12 software and cross-checked the data for accuracy. Coding was then conducted in three cycles. First, open coding was conducted; we summarized the raw qualitative data in words or short phrases. Second, focused coding was used to re-examine codes identified in the initial coding and develop different categories (Appendix C). Third, axial coding was conducted by reconfiguring the previous coding into three refined themes (Appendix C) based on RQ3 and RQ4 to search for rules, causes, and explanations in the data. The questionnaire results were translated from Chinese (the EFL medical students’ native language) into English; as mentioned above, the interviews were conducted in English.

## 4. Results and Discussion

### 4.1. RQ1: What Types of WCF Do EFL Medical Students Prefer to Receive?

**Figure 1:**
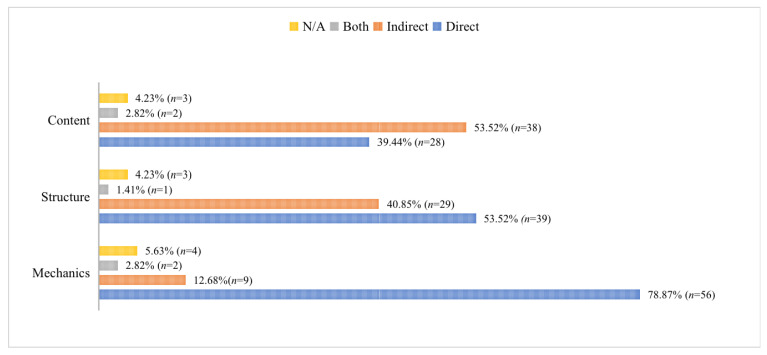
Students’ Preferences for Direct and Indirect Feedback. Note: N/A refers to missing answers.

Firstly, the results showed EFL medical students preferred indirect feedback on writing content but direct feedback for both writing mechanics and structure (Figure 1). EFL medical students preferred indirect feedback for writing content as it provided them with the opportunity to think over and revise the content. Contrastingly, as writing mechanics and structure are more teachable skills and require less creativity [35], EFL medical students preferred direct feedback. Gass’s [36] theoretical model of second language acquisition (SLA) explains that the learning process begins when learners attend to the target feature and notice the mismatch between the WCF input and their output. Then, learners need to apprehend the WCF input—either implicit or explicit—that has been noticed, in order for it to function as a noticing facilitator [37]. However, the degree of explicitness of WCF affects the way feedback is noticed; more explicit WCF types may lead to a higher level of noticing [37]. Moreover, although some previous studies have found no statistically significant differences between direct and indirect WCF [10], other researchers have asserted that direct feedback is more helpful in improving the accuracy of students’ writing over time [25].

**Figure 2:**
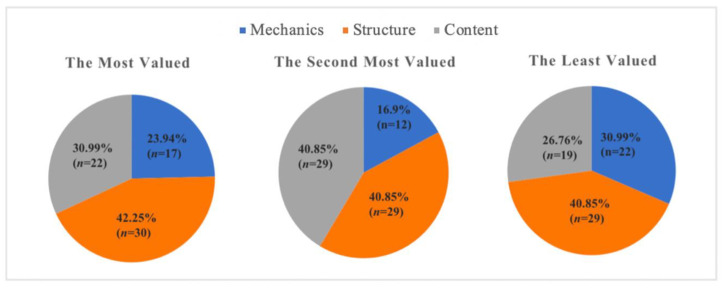
Students’ Preferences for Feedback on Writing Mechanics, Structure, and Content.

Secondly, the results showed that EFL medical students preferred to receive teacher feedback on their writing structure rather than their writing content or mechanics (Figure 2). By providing detail on appropriate structuring of content, writing structure feedback clarified students’ ideas about effectively articulating writing content. In contrast to Diab [38]—who found that EFL students rated teachers’ writing structure and content feedback equally—we found that most Taiwanese EFL medical students preferred writing structure feedback. As EFL medical students are required to write medical reports and patient histories, they need to follow specific writing structures; therefore, they may have valued feedback on writing structure more highly. Table 1 presents examples of the teacher’s WCF on students’ writing.

### 4.2. RQ2: What Types of WCF do EFL Medical Students Perceive as Helpful?

**Figure 3:**
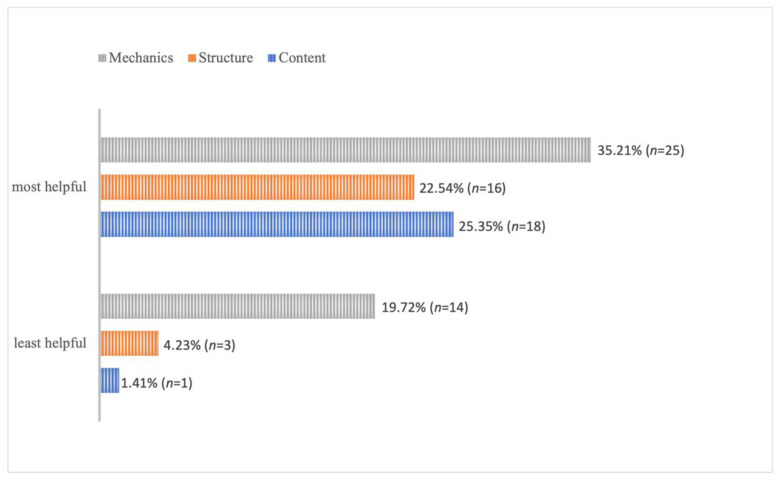
Students’ Perceptions of the Most and Least Helpful Feedback.

In response to question 5 (Appendix A), students reported both helpful and unhelpful teacher WCF (Table 2); examples of the participants’ comments are included in Appendix D. Compared to the structure and content, feedback on writing mechanics was perceived by more EFL medical students (*n* = 22) as the most helpful (Figure 3). Interestingly, writing mechanics was also chosen by more students (*n* = 14) as the least helpful. We speculate that writing mechanics feedback was chosen the most frequently for both helpful and unhelpful examples due to its relevance to teaching techniques in this context. Specifically, we attempt to explain the contradictory results as follows. Feedback on writing mechanics may have been perceived as the most helpful because it can simply be followed regardless of WCF type. Students also reported that feedback relating to spelling, punctuation, and particularly formatting (e.g., American Psychological Association (APA) style) is useful for their future studies. Contrastingly, feedback on writing mechanics may have been seen as the least helpful among students already familiar with conventions such as the APA style. These students may think writing mechanics feedback is unsophisticated, easy to correct, and less relevant than other forms.

### 4.3. RQ3: Why do EFL Medical Students Prefer Certain Types of WCF?

The interview data revealed three influencing factors of the students’ preferences, including student–teacher interactions, students’ self-expression, and L2 writing instruction. 

Firstly, as excerpt 1 below illustrates, student–teacher interactions may influence EFL medical students’ WCF preferences because EFL medical students preferred to receive indirect WCF, which requires more student–teacher interactions.

Excerpt 1

Teacher: The only thing the teacher can do is to try to guess or infer what the student was thinking when they completed the writing. If the teacher goes through the trouble of rewriting or giving feedback from the wrong perspective, then the practice of giving the feedback is kind of wasted. This is because it is not actually reformulating the student’s writing in the way that the student intended it.

Therefore, student–teacher interactions affect the way teachers impart and students understand feedback. Zhang [13] demonstrated that learners’ awareness of teachers’ feedback intention might influence their perceptions of the effectiveness of particular WCF types, highlighting the importance of considering learners’ preferences when providing WCF. Additionally, investigating students’ beliefs about the effect of WCF types, Diab [38] compared ESL students’ preferences for WCF and their interactions with teachers, revealing a conflict between learners’ and teachers’ views. Diab [38] concluded that teachers should attempt to understand students’ beliefs about WCF to connect their expectations with those of the students. In summary, the divergence between students’ and teachers’ preferences for WCF types can lead to inefficient WCF instruction; teacher WCF practices need to be considered as they may affect students’ perceptions of WCF’s usefulness [24]. Student–teacher interactions should be valued by teachers when providing WCF. For example, teachers should communicate with the students more often to discuss writing and provide feedback accordingly. More communication would help teachers understand students’ perceptions and beliefs about WCF and create a better connection between their expectations.

Secondly, EFL medical students’ self-expression may influence their WCF preferences. As mentioned above, EFL medical students preferred feedback on writing structure over writing content or mechanics, as it helped them clarify the expression of their ideas.

Excerpt 2

Teacher: It may be because they are trying to express their own opinions or content. If the teacher told them exactly what to write, then that’s a direct comment telling them what to do. They might feel they are losing their voices. … Students may also prefer [writing content feedback] because they probably think that content is the thing that teachers can correct … they can try to learn something from that correction. 

Excerpt 2 illustrates that EFL medical students’ self-expression may have influenced their WCF preferences. Our finding—that feedback on writing structure helped students clarify the expression of their ideas—suggests that such feedback may further encourage learner reflection and self-editing [39]. When providing writing structure feedback, the teacher tried to give some examples of topic, supporting, and closing sentences to clarify how the content could be organized. By following this feedback, students could adjust their writing independently, clarifying and improving the content.

Thirdly, EFL medical students’ WCF preferences may have been related to the teacher’s L2 writing instruction.

Excerpt 3

Teacher: I tried to help them get prepared to do the writing. After reading one article about the topic, I would always get students in groups for different kinds of discussion activities. They would talk about the topic before I would share my opinions with them. These brainstorming activities in class aimed at preparing them for the writing.

As excerpt 3 shows, the teacher guided the students in brainstorming about writing content, which may be why the EFL medical students preferred indirect WCF. The teacher ran a student-centered writing class that emphasized student autonomy. This learner agency could have familiarized them with processing indirect feedback. Therefore, EFL medical students’ WCF preferences may be relevant to the teacher’s L2 instruction in writing classes. Rahimi [40] indicated that students’ beliefs about different grammatical units were shaped by the teacher’s practice and his emphasis on certain WCF strategies. Additionally, the results showed that students did not consider direct WCF very helpful when essays did not need to be revised subsequently [40]. Students also thought that self-editing and indirect WCF were illogical and unhelpful strategies [40]. Séror’s [41] findings revealed that students preferred longer WCF comments on their ideas, thoughts, arguments, and content, not just WCF focusing on grammar. Séror [41] also described how learners found handwritten indirect WCF difficult to decipher. Furthermore, students reported that this style of WCF neither addressed their writing problems nor provided specific solutions [41]. 

### 4.4. RQ4: Why do EFL Medical Students Perceive Certain Types of WCF as Helpful and Others as Unhelpful?

Explaining why EFL medical students perceived certain types of WCF as helpful, both perceived benefits and challenges were detected in the interview data (Table 3). Perceived benefits included language development, writing improvement, peer comparison, and emotional satisfaction. Specifically, EFL medical students perceived certain WCF types as helpful because they facilitated an improvement in their English writing ability, encouraged healthy competition, and provided positive emotional support (e.g., [24,25,26]. Perceived WCF challenges included heavy workload, inadequate comments, unclear policy, and ineffective communication. Specifically, EFL medical students perceived certain WCF types as unhelpful because they increased the workload without improving students’ writing. Additionally, inadequate teacher comments added to their confusion about correct writing. The other two reasons were related to unclear school policy regarding EFL medical students’ learning of English writing and ineffective communication between medical teachers and language teachers. A clear school policy and effective communication among teachers facilitates students’ learning and teachers’ teaching. However, this medical school’s current policy is unclear in terms of medical students’ English writing requirements. The English language curriculum required satisfactory completion of four credits of general English language classes and two credits of academic English. English language education was seen as a part of general education, but had no specific curriculum. Therefore, English teachers were only required to follow basic guidelines for teaching and evaluation. Additionally, there was almost no communication between the medical school professors and the English language teachers; this was part of two perceived challenges to English writing improvement among EFL medical students.

Previously, Bailey [42] found that participants preferred more extensive help over small condensed WCF, such as exclamation symbols in the margin; learners perceived elaborate WCF as offering a superior learning experience that increased their general and essay-writing skills. Furthermore, Bailey [42] revealed that learners were dissatisfied not only with insufficient WCF, but also with the teachers’ inadequate comments. Moreover, Zhou [43] investigated ESL students’ motivations and strategies for improving their written grammar and vocabulary in a pre-university English for Academic Purposes course; the findings suggested teachers raise their learners’ awareness of achievable goals and help identify learners’ grammar difficulties. Aridah et al. [28] also investigated learners’ WCF-type preferences and found some discrepancies between students’ preferences and teachers’ practices. Although Aridah et al. [28] revealed that both student and teacher participants preferred direct WCF, students preferred to have more direct unfocused WCF, whereas teachers provided more indirect WCF than students expected. These findings led the authors to develop a preference-based WCF model to profile students’ preferences and teachers’ practices; our findings lead us to agree with the suggestion for a preference-based model of WCF.

## 5. Conclusions

In conclusion, the results showed that students preferred WCF on writing structure. Additionally, they preferred direct over indirect feedback for both writing mechanics and structure. Compared to structure and content, specific instances of feedback on writing mechanics were perceived as both the most and least helpful. Two in-depth interviews uncovered the factors influencing EFL medical students’ preferences and perceived WCF-related benefits and challenges. 

In light of EFL medical students’ specialized perceptions and preferences, we now discuss the pedagogical implications, with a focus on L2 writing teachers’ feedback-providing strategies related to students’ writing content and structure. Specifically, we suggest teachers provide more effective comments on students’ English academic writing. Furthermore, EFL medical students expected to receive more emotional support, especially positive encouragement, from their teachers and to establish effective communication. WCF offers teachers an opportunity to examine, through reflection and practitioner research, a specific aspect of their own instructional practices, and, in so doing, to contribute to our general understanding of how WCF can be most effectively executed to promote language learning. Overall, the findings support pedagogical approaches that systematically incorporate teacher WCF for EFL medical students. We suggest L2 writing teachers use automated writing evaluation for writing mechanics and grammar feedback, freeing up their time to provide in-depth feedback on writing content and structure. 

Handling the complexities of WCF is a key pedagogical issue. Clearly, simplistic pedagogical proscriptions and prescriptions no longer reflect the reality of either the process by which WCF is enacted or its student performance outcomes. Accordingly, this study suggests that teacher education programs present teachers with a set of guidelines that can serve as a basis for reflection and teacher-led research into WCF. Teachers need to be guided by research but also need to establish the extent to which its findings are applicable to their own classrooms.

Several limitations to this intrinsic case study need to be stated. First, the participants were recruited from a large medical university in Taiwan; thus, the findings may only have direct relevance for the EFL medical English program at this university or other universities with a similar institutional context. Second, as this study only involved EFL medical students, the results may not represent the learning situations of EFL students in other majors. Third, another limitation is that we did not conduct follow-up interviews with the students. Thus, we suggest that future studies continue to shed light on this research area with a larger sample size in a variety of institutional contexts.

## Figures and Tables

**Table 1:** Examples of the Teacher’s Written Corrective Feedback.

Types	Examples
Direct feedback for content	“You have written that *women tend to have longer life expectancy than men*. You should provide some details to support this statement and explain why this might be.”
Direct feedback for structure	“In this paragraph you should have explained why life expectancy is generally increasing throughout the world and then in the following paragraph you should have used the information from your research article(s) to explain what is happening to life expectancy in the country you selected.”
Direct feedback for mechanics	“This is the first use of the acronym WHO, [and] so you should write it out in full as World Health Organization (WHO). All subsequent mentions can use the abbreviation WHO.”
Indirect feedback for content	“I can’t understand the cause and effect relationship that you are trying to make here. Consider rewriting/revising this sentence.”
Indirect feedback for structure	“Should this be where you describe the disease?”
Indirect feedback for mechanics	“In 1967, the WHO became concered with the eradication ^a^ of smallpox.”

Note: ^a^ This is indirect feedback regarding the spelling of “concerned” and “eradication”.

**Table 2:** Students’ Perceptions of Writing Mechanics Feedback.

The Most Helpful	The Least Helpful
➢ Direct correction (*n* = 10) ➢ Direct corrections with examples or explanations (*n* = 3) ➢ Suggestions (*n* = 3) ➢ Encouragement (*n* = 3) ➢ Open-ended questions (*n* = 3)	➢ Open-ended questions (*n* = 6) ➢ Indirect feedback or general comments without details, examples, or explanations (*n* = 5) ➢ Unclear handwriting (*n* = 2) ➢ Provide scores (*n* = 1)

Note: While there were 71 participants, not all provided answers for certain questions.

**Table 3:** Students’ Perceived Benefits and Challenges Related to WCF.

Benefits	Challenges
➢ Language development ➢ Writing improvement ➢ Peer comparison ➢ Emotional satisfaction	➢ Heavy workload ➢ Inadequate comments ➢ Unclear policy ➢ Ineffective communication

## Data Availability

The data presented in this study are available on request from the corresponding author. The data are not publicly available due to restrictions set by the ethics committee review.

## Appendix A

**Written Corrective Feedback Questionnaire (English version)**

**Name:**

Teacher feedback refers to the teacher providing comments, corrections, suggestions, and questions for students to revise and reflect on their writing. This questionnaire includes questions on three aspects of feedback: mechanics (grammar and vocabulary), structure (structure, transitions, and paraphrasing), and content (ideas, arguments, and concepts).

1. Please explain how you feel about the writing feedback given by the teacher. Do you like it or dislike it? Do you think it helped? Please think about what kind of feedback you like and which kind of feedback you dislike. Please express your feelings in as much detail as possible.
2. Please answer the following questions based on your preferences for teacher written corrective feedback.

2a. Writing mechanics: please circle your preference

Direct feedback

I were was going to the store.

Indirect feedback

I were going to the store. How do you form the past progressive? Consider your auxiliary verb.

2b. Writing structure: please circle your preference

Direct feedback

You need to break your paragraph after the fifth sentence.

Indirect feedback

Take a look at your third paragraph and read over it. Do you notice where your tone changes? Could this be a paragraph break?

2c. Writing content: please circle your preference

Direct feedback

This idea is not strong enough. It should be replaced with research.

Indirect feedback

Consider how your audience might react to this conclusion. Does your evidence support it? How could you make your supporting points stronger?

3. Based on your opinions and experience, please rate the importance of the following feedback types:

1 = You think the feedback is the most important; 2 = You think the feedback is second most important; 3 = You think the feedback is the least important

    1. mechanics structure content

    2. mechanics structure content

    3. mechanics structure content

4. Please explain your choice based on the above question, or give other comments about writing feedback.

4a. What kind of feedback is most helpful to you? Please explain in detail.

4b. What kind of feedback is least helpful to you? Please explain in detail.

4c. For the following questions, please circle the response that best represents your opinion.

5. Do you value teachers’ feedback on concepts or ideas? Yes/No/No comment
6. Do you value teachers’ feedback on grammar? Yes/No/No comment
7. What kind of feedback do you value? Content or Idea/Grammar/Both
8. Have your thoughts about written corrective feedback changed after taking this course? If so, please explain.

**寫作回饋問卷 (Chinese Version)**

姓名：

教師回饋是由老師給予評論、糾正、建議以及問題來讓學生進行修改以及反思。該問卷包含三項回饋的範疇：技巧(文法及字彙)、架構(結構、轉折及改寫)以及內容(想法、論證及概念)。

一、請說明你對老師所給予的寫作回饋有何感受呢？你喜歡還是討厭呢？你覺得有沒有幫助呢？請你想想哪種回饋是你喜歡的，而哪種是你討厭的？請盡可能發表你的感受。

二、請根據你對於寫作回饋的喜好，回答下列問題。

1. 技巧回饋：請圈選你喜愛的回饋

清楚糾正

I were was going to the store.

問題/建議

I were going to the store. *How do you form the past progressive? Consider your auxiliary verb.*

2. 架構回饋：請圈選你喜愛的回饋

清楚糾正

*You need to break your paragraph after the fifth sentence.*

問題/建議

*Take a look at your third paragraph and read over it. Do you notice where your tone changes? Could this be a paragraph break?*

3. 內容回饋：請圈選你喜愛的回饋

清楚糾正

*This idea is not strong enough. It should be replaced with research.*

問題/建議

*Consider how your audience might react to this conclusion. Does your evidence support it? How could you make your supporting points stronger?*

三、根據你的看法及經驗，請根據下列回饋種類評定其重要性：

1 = 你認為最重要的回饋；2 = 你認為次重要的回饋；3 = 你認為最不重要的回饋

    1. 技巧 架構 內容

    2. 技巧 架構 內容

    3. 技巧 架構 內容

四、根據上題請說明你的選擇，或是針對寫作回饋給予其它評論。

五、請參照你在課堂上所接收的回饋。根據你自己的意見回答下列問題。

什麼種類的回饋對你來說最有幫助？請詳細說明。

什麼種類的回饋對你來說是最沒有幫助的？請詳細說明。

六、請根據下列問題圈選最符合你意見的回應。

你重視教師在概念或想法上的回饋嗎？ 是 否 沒意見

你重視教師在文法上的回饋嗎？ 是 否 沒意見

你重視哪種回饋呢？      概念/想法 文法 两者

七、在經過本學期的課程後，你對於寫作回饋的想法有所改變嗎？如果有，請說明。

## Appendix B

**First semi-structured interviews questions**

1. Could you please introduce the academic “reading to write” course that you taught?
2. Did you provide writing feedback to your students?
3. When and how did you provide writing feedback to your students?
4. What types of writing feedback did you provide in the course?
5. Why did you provide certain types of writing feedback?
6. What types of writing feedback did the students prefer to receive?
7. Why did they prefer certain types of writing feedback?
8. When receiving teacher feedback in the classroom, what aspects did the students like most and why?
9. When receiving teacher feedback in the classroom, what aspects did students dislike most and why?
10. Do you think students’ thoughts about WCF changed after taking that course? Why or why not?

**Second semi-structured interviews questions**

1. Could you provide some basic information about the school’s English program?
2. How do you feel about the school’s policy about English language education?
3. How do you feel about the school’s curriculum and instruction about English language education?
4. Do you think this group of students are involved in a unique English language learning situation?
5. Why did the EFL medical students prefer certain types of WCF?
6. Why did the EFL medical students prefer indirect feedback on writing content?
7. Why did the EFL medical students prefer direct feedback over indirect feedback for writing mechanics?
8. Why did the EFL medical students prefer direct feedback over indirect feedback for writing structure?
9. Why did the EFL medical students perceive certain types of WCF as effective or ineffective?
10. What kind of feedback was most helpful or effective for EFL medical students?
11. What kind of feedback was least helpful or ineffective for EFL medical students?
12. Did the EFL medical students show you positive or negative feelings about the WCF?

## Appendix C

**Coding categories**	**Abbreviation**
Category 1: Student-teacher interactions	STI
Category 2: Students’ self-expression	SS
Category 3: Second language writing instruction	SLWI
Category 4: Language development	LD
Category 5: Writing improvement	WI
Category 6: Peer comparison	PC
Category 7: Emotional satisfaction	ES
Category 8: Heavy workload	HW
Category 9: Inadequate comments	IC
Category 10: Unclear policy	UP
Category 11: Ineffective communication	ICN
**Coding themes**	**Abbreviation**
Theme 1: Influencing factors of students’ preferences	IFSP
Theme 2: Perceived benefits from the WCF	PB
Theme 3: Perceived challenges from the WCF	PC

## Appendix D

**Examples of Students’ Comments on Writing Mechanics Feedback**

**The most helpful feedback on writing mechanics**

1. Direct correction (*n* = 10)
  Example: “Direct pointing out the grammatical and content faults.”
2. Direct corrections with examples or explanations (*n* = 3)
  Example: “Direct corrections of our problems with some examples as references.”
3. Suggestions (*n* = 3)
  Example: “The teacher provides us many suggestions on the grammar and sentence. During high school time, we practice the diversification of sentences by English writing exercises, for instance, inversion of a sentence and participle clause etc.. But it is not the same in academic English writing. It takes long to refine complicated sentences and prepositions.”
4. Encouragement (*n* = 3)
  Example: “In the way of encouragement.”
5. Open-ended questions (*n* = 3)
  Example: “The feedback in the way of reflection to our ideas and rhetorical questions are of great help, which can inspire me to think more deeply.”

**The least helpful feedback on writing mechanics**

1. Open-ended questions (*n* = 6)
  Example: “Open-ended questions confuse me a lot.”
2. Indirect feedback or general comments without details, examples, or explanations (*n* = 5)
  Example: “General descriptions without details.”
3. Unclear handwriting (*n* = 2)
  Example: “I cannot figure out what the teacher writes because of the unclear handwriting.”
4. Provide scores (*n* = 1)
  Example: “The teacher only gives us the score but doesn’t explain strengths and weaknesses of our writing.”

## References

[1] Amrhein H.R., Nassaji H. (2010). Written corrective feedback: What do students and teachers think is right and why?. Can. J. Appl. Linguist..

[2] Sarkeshikian A., Lotfi S.A.T., Hayali F. (2020). Written corrective feedback across disciplines: A case of PhD candidates’ perceptions and preferences. Appl. Linguist. Res. J..

[3] Bitchener J., Ferris D.R. (2021). Written Corrective Feedback in Second Language Acquisition and Writing.

[4] Truscott J. (1996). The case against grammar correction in L2 writing classes. Lang. Learn..

[5] Ferris D.R., Hyland K., Hyland F. (2006). Does error feedback help student writers? New evidence on the shortand long-term effects of written error correction. Feedback in Second Language Writing: Contexts and Issues.

[6] Lim S.C., Renandya W.A. (2020). Efficacy of written corrective feedback in writing instruction: A meta-analysis. TESL-EJ.

[7] Ellis R. (2010). Epilogue: A framework for investigating oral and written corrective feedback. Stud. Second Lang. Acquis..

[8] Ellis R. (2009). Corrective feedback and teacher development. L2 J..

[9] Lee I. (2008). Student reactions to teacher feedback in two Hong Kong secondary classrooms. J. Second. Lang. Writ..

[10] Chen S., Nassaji H., Liu Q. (2016). EFL learners’ perceptions and preferences of written corrective feedback: A case study of university students from Mainland China. Asian-Pac. J. Second Foreign Lang. Educ..

[11] Alshahrani A., Storch N. (2014). Investigating teachers’ written corrective feedback practices in a Saudi EFL context: How do they align with their beliefs, institutional guidelines, and students’ preferences?. Aust. Rev. Appl. Linguist..

[12] Lee I. (2005). Error correction in the L2 writing classroom: What do students think?. TESL Can. J..

[13] Zhang Z. (2020). Engaging with automated writing evaluation (AWE) feedback on L2 writing: Student perceptions and revisions. Assess. Writ..

[14] Zhang Z., Hyland K. (2018). Student engagement with teacher and automated feedback on L2 writing. Assess. Writ..

[15] Rummel S., Bitchener J. (2015). The effectiveness of written corrective feedback and the impact Lao learners’ beliefs have on uptake. Aust. Rev. Appl. Linguist..

[16] Barrot J.S. (2021). Using automated written corrective feedback in the writing classrooms: Effects on L2 writing accuracy. Comput. Assist. Lang. Learn..

[17] Bing-You R., Varaklis K., Hayes V., Trowbridge R., Kemp H., McKelvy D. (2018). The feedback tango: An integrative review and analysis of the content of the teacher–learner feedback exchange. Acad. Med..

[18] Ha X.V., Nguyen L.T., Hung B.P. (2021). Oral corrective feedback in English as a foreign language classrooms: A teaching and learning perspective. Heliyon.

[19] Mujtaba S.M., Reynolds B.L., Parkash R., Singh M.K.M. (2021). Individual and collaborative processing of written corrective feedback affects second language writing accuracy and revision. Assess. Writ..

[20] Long M., Ritchie W.C., Bhatia T.K. (1996). The role of the linguistic environment in second language acquisition. Handbook of Second Language Acquisition.

[21] DeKeyser R., Doughty C., Williams J. (1998). Beyond focus on form: Cognitive perspectives on learning and practicing second language grammar. Focus on Form in Classroom Second Language Acquisition.

[22] Ferris D. (1999). The case for grammar correction in L2 writing classes: A response to Truscott (1996). J. Second Lang. Writ..

[23] Lee I. (2017). Classroom Writing Assessment and Feedback in L2 School Contexts.

[24] Zhang T., Chen X., Hu J., Ketwan P. (2021). EFL students’ preferences for written corrective feedback: Do error types, language proficiency, and foreign language enjoyment matter?. Front. Psychol..

[25] Bitchener J., Knoch U. (2010). Raising the linguistic accuracy level of advanced L2 writers with written corrective feedback. J. Second Lang. Writ..

[26] Guo Q., Barrot J.S. (2019). Effects of metalinguistic explanation and direct correction on EFL learners’ linguistic accuracy. Read. Writ. Q..

[27] Saeli H., Cheng A. (2019). Student writers’ affective engagement with grammar-centred written corrective feedback: The impact of (mis) aligned practices and perceptions. Can. J. Appl. Linguist. Rev. Can. Linguist. Appliquée.

[28] Aridah A., Atmowardoyo H., Salija K. (2017). Teacher practices and students’ preferences for written corrective feedback and their implications on writing instruction. Int. J. Engl. Linguist..

[29] Diab N.M. (2015). Effectiveness of written corrective feedback: Does type of error and type of correction matter?. Assess. Writ..

[30] Chu H.Y., Yeh H.N. (2017). English benchmark policy for graduation in Taiwan’s higher education: Investigation and reflection. J. Lang. Teach. Res..

[31] Wu M. (2012). Comparing PETS and GEPT in China and Taiwan. Engl. Lang. Teach..

[32] Reynolds B.L., Teng M.F. (2021). Innovative Approaches in Teaching English Writing to Chinese Speakers.

[33] Ellis R. (2009). A typology of written corrective feedback types. ELT J..

[34] Creswell J.W., Hanson W.E., Clark Plano V.L., Morales A. (2007). Qualitative research designs: Selection and implementation. Couns. Psychol..

[35] Alpert F., Antonangeli I., Cremins A., Doon L., Genovesi L., Lewis S.C., McGilvery H.L., Rothblatt H., Stevens R. (1956). Effect of Proofreading on Mechanics and Structure of Writing—Grades Four, Five, Six. Master’s Dissertation.

[36] Gass S. (1997). Input, Interaction, and the Second Language Learner.

[37] Shintani N., Ellis R. (2013). The comparative effect of direct written corrective feedback and metalinguistic explanation on learners’ explicit and implicit knowledge of the English indefinite article. J. Second Lang. Writ..

[38] Diab R.L. (2005). Teachers’ and students’ beliefs about responding to ESL writing: A case study. TESL Can. J..

[39] Hyland K., Hyland F. (2006). Feedback on second language students’ writing. Lang. Teach..

[40] Rahimi M. (2012). Iranian EFL students’ perceptions and preferences for teachers’ written feedback: Do students’ ideas reflect teachers’ practice?. J. Teach. Lang. Ski..

[41] Séror J. (2009). Institutional forces and L2 writing feedback in higher education. Can. Mod. Lang. Rev..

[42] Bailey R.A. (2009). Undergraduate students’ perceptions the role and utility of written assessment feedback. J. Learn. Dev. High. Educ..

[43] Zhou A.A. (2009). What adult ESL learners say about improving grammar and vocabulary in their writing for academic purposes. Lang. Aware..

